# A Case Report of an Atypical Presentation of Fournier’s Gangrene

**DOI:** 10.5070/M5.52203

**Published:** 2026-01-31

**Authors:** Elaha Noori, Konnor Davis, Tyler Rigdon, Lindsey Spiegelman

**Affiliations:** * University of California, Irvine, School of Medicine, Irvine, CA; ^ University of California, Irvine, Department of Emergency Medicine, Orange, CA

## Abstract

Fournier’s gangrene is a well-established surgical emergency as patients can decompensate rapidly, resulting in significant morbidity and mortality. We describe a case in which a 54-year-old medically complex male presented to the emergency department (ED) with a primary complaint of abdominal pain after a therapeutic paracentesis was performed by interventional radiology just prior to arrival. Upon further questioning, the patient reported scrotal pain that had developed over three to four days. On exam, the patient was well-appearing although he did have scattered scrotal masses with chalky white purulence. Given the relatively quick development, an atypical presentation of Fournier’s gangrene was suspected, and the patient was started on antibiotics with urology immediately consulted. Imaging was concerning for subcutaneous gas. The patient was subsequently taken to the operating room (OR) for debridement, where Fournier’s gangrene was confirmed by surgical investigation and culture. Significant lessons from the case include the importance of early identification of Fournier’s gangrene and having a low threshold to start treatment in coordination with a surgical service for atypical presentations.

**Topics:**

Fournier’s gangrene, bilateral epididymitis, scrotal pain, urologic emergency, renal transplant.

**Figure:**
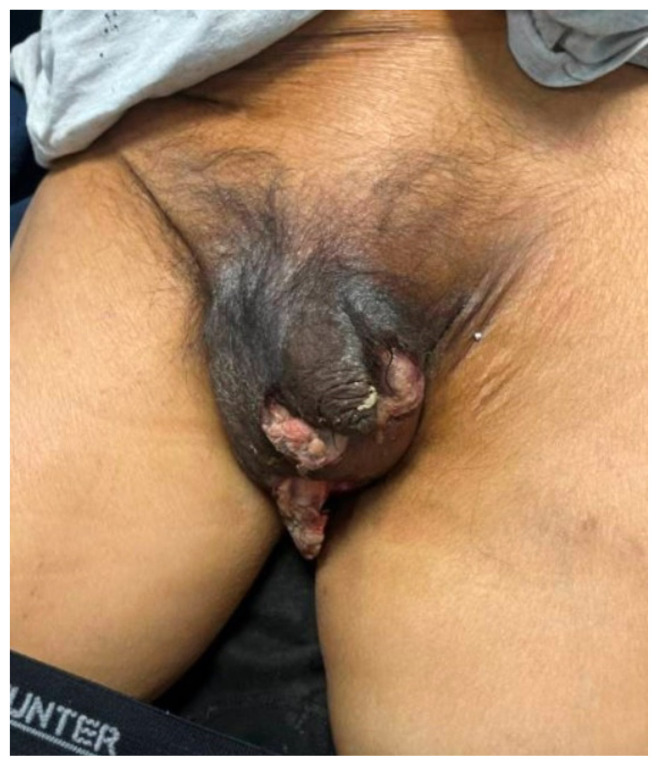


**Figure:**
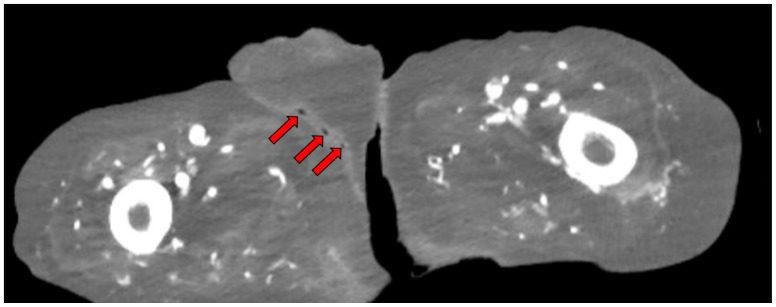


## Brief introduction

Fournier’s gangrene is a rare, fulminant form of necrotizing soft tissue infection involving the genital, perineal, or perianal regions. Due to the high degree of rapid disease progression and death, it is a surgical emergency that is important for emergency medicine physicians to identify on initial evaluation.^1^ Fournier’s gangrene is known to be polymicrobial in nature, but the most common isolated organisms include *Escherichia coli*, *Streptococcus*, and *Pseudomonas*. Urinary tract, colorectal, and anorectal regions are typical portals of entry that allow the infection to spread into the deep fascia and cause progressive necrosis of tissue. Although recognized to occur in both males and females of any age, Fournier’s gangrene shows a male dominance with other risk factors including diabetes mellitus, chronic alcoholism, obesity, immunosuppression, and age greater than 50 years old.^2^,^3^ Classically, Fournier’s gangrene is characterized by severe pain that starts in the anterior abdominal wall and travels to urogenital and anogenital areas, with early clinical features including fever, chills, perineal swelling, and erythema. However, more specific signs of crepitus, bullae, and subcutaneous gas often present much later and therefore present a challenge in the rapid diagnosis of Fournier’s gangrene in the acute clinical setting.^4^ Management includes emergency debridement, administration of broad-spectrum intravenous antibiotics, and hemodynamic support.

Fournier’s gangrene is a life-threatening necrotizing infection that requires early diagnosis and intervention to reduce mortality and improve patient outcomes. Here, we present the case of a 54-year-old medically complex male who presented with a distracting initial complaint of abdominal pain after a paracentesis. However, on further questioning, he was found to have scrotal pain concerning for an atypical presentation of Fournier’s gangrene.

## Presenting concerns and clinical findings

A 54-year-old male with a past medical history of hypertension, coronary artery disease, *Clostridioides difficile (C. Diff*) infections, type 2 diabetes mellitus (T2DM), end-stage renal disease (ESRD) status post deceased donor kidney transplant, and cirrhosis presented to the ED with abdominal pain after a therapeutic paracentesis (removing three liters) was performed by interventional radiology just prior to arrival. Upon further questioning, the patient stated that his pain was lower in his scrotum and had progressively developed over the past three to four days. He also reported a wound on his scrotum and associated dysuria. The patient denied fevers, rash, chest pain, back pain, vomiting, or diarrhea. His initial vital signs included an elevated blood pressure of 209/141 mmHg, a pulse of 69 beats per minute, a temperature of 97.7°F, a respiratory rate of 20 breaths per minute, and blood oxygen saturation of 100% on room air. Upon physical examination, the patient’s abdomen was soft and nontender with mild distension. There was no guarding or rigidity. The genitourinary examination indicated edema and severe tenderness to palpation to the scrotum bilaterally, with scattered ulcerations, skin breakdown, and chalky white purulence present on the scrotum. The testicles had a vertical lie, and there was no palpable crepitus, rash, perineal involvement, or penile discharge. Physical examination was otherwise unremarkable. Written consent was obtained from the patient for photograph and image publication.

The patient’s ESRD was thought to be due to T2DM and was treated with hemodialysis for nine years before receiving a transplant in January 2019. A biopsy in 2022 demonstrated interstitial fibrosis, tubular atrophy, and antibody mediated rejection. Unfortunately, he had not received immunosuppression for over a year at the time of biopsy due to financial obstacles. The patient’s liver cirrhosis was recently diagnosed and has been receiving interventional radiology-guided paracenteses for recurrent ascites since April. Besides financial concerns, the patient presented with no significant social or family history. Surgical history was significant for left below the knee amputation in 2023 for osteomyelitis of prior transmetatarsal amputation.

## Significant findings

The clinical image of the patient’s case demonstrated marked scrotal swelling with overlying black induration extending to the penis, consistent with advanced cutaneous necrosis. Scattered ulcerations and areas of skin breakdown were visible, with chalky white purulent material expressed from the surface. The clinical presentation was highly characteristic of a necrotizing infection.

The patient’s workup in the ED included labs, computed tomography (CT) scans, ultrasound imaging, and a urology consult. Labs were remarkable for an elevated white blood cell count of 11.3 x 10^9^/Liter, low hemoglobin at 9.5 grams per deciliter (g/dL), low serum sodium at 128 milliequivalents per liter (mEq/L), elevated blood urea nitrogen at 99 milligrams per deciliter (mg/dL), and elevated creatinine at 4.2 mg/dL. Additionally, erythrocyte sedimentation rate and serum C-reactive protein levels had marked elevations at 56 millimeters per hour (mm/hr) and 5.8 mg/dL, respectively. Urinalysis was indicative of pyuria with positive leukocyte esterase, an elevated white blood cell count of 182 per high-power field, proteinuria, and presence of a few bacteria. Glucose and lactic acid were within normal limits. Given the patient’s physical exam and lab findings, urology was immediately consulted due to concern for a necrotizing soft tissue infection. While waiting for imaging to result, the patient’s laboratory risk indicator for necrotizing fasciitis (LRINEC) score was calculated to be 6, which indicated intermediate risk.^5^

A computed tomography (CT) scan of the abdomen and pelvis, recommended by urology, was significant for scrotal fluid and punctate gas locules (red arrow) without discrete evidence of invasion into the adjacent soft tissues, suspicious for Fournier’s gangrene. There was also fluid collection centered around the seminal vesicles suggestive of an abscess. Additionally, a doppler ultrasound showed bilateral epididymo-orchitis and suspected subcutaneous emphysema of the left scrotal wall, while a duplex ultrasound revealed normal arterial and venous flow in the bilateral testes.

## Patient course

Overall, the patient’s rapid progression of acute scrotal pain, severe tenderness out of proportion to findings, and necrotic skin changes were most consistent with Fournier’s gangrene. The presence of cutaneous necrosis and purulent drainage was evidence of deeper fascial involvement and pointed away from superficial scrotal cellulitis. Alternative diagnoses such as inguinoscrotal abscess and epididymo-orchitis were considered due to the CT and ultrasound findings of fluid collection. However, these conditions alone would not account for the diffuse cutaneous necrosis and presence of gas, which were more specific for Fournier’s gangrene. Malignancy was much less likely due to the acute onset and lack of systemic symptoms (eg, fever, weight loss, night sweats). Testicular torsion was effectively excluded due to the normal testicular lie and preserved vascular flow on duplex ultrasound.

Given the patient’s initial presentation and physical exam findings, a necrotizing soft tissue infection was suspected. The patient was promptly started on broad-spectrum antibiotics (intravenous vancomycin, piperacillin/tazobactam, and clindamycin) with intravenous acetaminophen for pain control. Hydralazine and nicardipine were also administered in the ED to control the patient’s persistently high blood pressure. Shortly after treatment was initiated, urology was consulted for immediate evaluation, and a CT scan with contrast of the abdomen and pelvis was ordered. Due to the patient’s history of kidney transplant, transplant nephrology was also contacted, and it was advised to administer normal saline at 75 milliliters per hour prior to and several hours after the CT scan to prevent contrast-induced nephropathy.

Upon receipt of the patient’s CT results, there was now further evidence of a necrotizing soft tissue infection of the scrotum in correlation with the patient’s clinical presentation. The patient was emergently taken to the OR where he underwent debridement by the urology surgical team and where wound cultures confirmed a polymicrobial infection including gram-negative bacilli and nonhemolytic streptococci. A second debridement occurred two days later.

Following the first debridement, the patient was admitted to the internal medicine team with a diagnosis of Fournier’s gangrene, complicated by extended-spectrum beta-lactamase-producing *Escherichia coli* confirmed by blood culture. He was thereafter started on meropenem, linezolid (in replacement of clindamycin), and oral vancomycin for *C. diff* prophylaxis as recommended by infectious disease (ID). Closer to discharge, the patient was transitioned to imipenem while continuing oral vancomycin. Due to poor renal function, imipenem was later switched to daptomycin and ertapenem. The patient had been off hemodialysis due to loss of medical insurance; however, renal failure during admission necessitated re-initiation of dialysis. Moreover, the patient had persistently low glucose levels (around 70 mg/dL) secondary to end stage renal disease and cirrhosis, but he improved significantly after transitioning from a diabetic diet to a regular diet.

During the patient’s inpatient course, he was also followed closely by urology, plastic surgery, wound care, ID, and even interventional radiology for therapeutic paracenteses. Evaluation by plastic surgery demonstrated no signs of healing within the wound bed, so reconstructive surgical interventions were not considered due to the high risk of failure and dehiscence given the patient’s comorbidities. Local wound care was instead continued with twice daily wet-to-dry gauze dressings in the hospital. During his hospital course, bilateral seminal vesicle abscesses with rectal fistulation were noted. Various consulting services declined immediate intervention due to presence of gas, poor surgical candidacy, and the fluid draining on its own. With the patient’s complicated ID course involving multidrug-resistant organisms and the seminal vesicle abscesses, ID recommendations upon discharge included four weeks of daptomycin and ertapenem treatment with oral vancomycin for *C. diff* suppression for one week after all other antibiotics were finished. On hospital admission day 33, the patient was discharged home with antibiotics and with established outpatient wound care.

## Discussion

Fournier’s gangrene, a morbid necrotizing soft tissue infection affecting the external genitalia and perineum, is a rare urologic emergency representing less than 0.02% of hospital admissions in the United States.^1^ Though previously considered idiopathic in origin, its etiology is typically a pathological process localized to urogenital and anorectal sources, including urinary tract infections, traumatic urogenital injuries, epididymitis, and perianal or perirectal abscesses. The infection often begins as cellulitis adjacent to the bacterial portal of entry. Cultures of wounds often demonstrate polymicrobial infections with both aerobes and anaerobes, with *Escherichia coli* being the most common isolated organism.^3^ As bacteria penetrate through the superficial and deep perineal fascia, this results in an inflammatory vascular thrombosis, tissue necrosis, and localized ischemia.^6^ If not treated aggressively, the infection rapidly progresses to sepsis and multiple organ failure, which are the most common causes of mortality.^7^

Current mortality rates vary widely. While a 2009 study utilizing a national inpatient database demonstrated an overall population-based fatality rate of 7.5%, other studies at individual tertiary care referral centers found higher mortality rates between 20 to 40%, with one center even reporting a mortality rate of 88%.^1^,^8^,^9^ These varying rates are attributed to institutional differences in the clinical volumes, referral rates, and early recognition and treatment of Fournier’s gangrene cases. Moreover, the prompt recognition of these cases is hindered by the wide variability in presentation. It has a clinical course that is challenging to predict, with cases varying from an indolent onset and slow progression to those with a rapid, fulminant course. Thus, the diagnosis of Fournier’s gangrene requires a high index of suspicion.

The most common predisposing risk factors of Fournier’s gangrene include a history of diabetes mellitus, alcoholism, obesity, and immunosuppressive states. Male to female ratio is 10:1, with peak age being between 50 and 79 years.^2^,^3^,^10^ A systematic review of 37 cases identified that the most common clinical presentations of Fournier’s gangrene included scrotal and labial pain, erythema, cellulitis, fever, abscesses, and crepitus. However, 40% of patients presented with no discernible symptoms and had an insidious onset.^11^ While diagnosis is primarily clinical, imaging modalities such as CT and ultrasound may be useful in atypical presentations. CT imaging remains the most sensitive (88.5%) and specific (93.3%) modality in identifying Fournier’s gangrene through findings of abnormal fluid collections and subcutaneous emphysema.^12^ Nevertheless, imaging should not delay prompt diagnosis and treatment if there is high clinical suspicion of Fournier’s gangrene. Tools such as the LRINEC score have been developed to aid in distinguishing between necrotizing fasciitis and other soft tissue infections, but they are limited due to their high false positive and high false negative rates when utilized in ED settings.^13^

In our case, we present a 54-year-old male with multiple comorbidities including T2DM, ESRD, and kidney transplant status who initially presented for abdominal pain after a therapeutic paracentesis but was later found to be complaining of scrotal pain. While the patient’s physical examination was significant for severe scrotal tenderness and edema, he did not display typical presenting signs of Fournier’s gangrene such as fever, crepitus, erythema, or perineal involvement. Previous atypical cases lacking these features have been reported in the literature, particularly among older, diabetic, and immunocompromised patients. For instance, Rahmatika et al described a 65-year-old male with scrotal pain who exhibited no signs of crepitus or necrotic changes despite x-ray findings of gas gangrene.^14^ Similarly, George et al detailed the case of a 57-year-old diabetic male who had presented without discernible crepitus despite ultrasound demonstrating gas in the scrotum.^15^ Both prior Fournier’s gangrene cases were devoid of hallmark signs of necrotizing infection on initial examination, underscoring that external skin findings may lag behind deep fascial involvement. Our patient’s presentation closely paralleled these atypical reports, in that his significant pain and rapid progression contrasted sharply with the absence of classic physical exam findings. This overlap highlights the importance of maintaining high clinical suspicion and utilizing adjunctive imaging to prevent life-threatening delays in diagnosis.

The strengths of this case report lie in the timely recognition of an atypical Fournier’s gangrene presentation, early involvement of the urology team, and treatment in a medically complex patient. There were many important steps taken to minimize morbidity, especially the first steps of initiating broad-spectrum antibiotics and consulting urology even prior to receiving further confirmation through CT imaging. Surgical debridement and broad-spectrum antibiotic therapy are gold standard treatments of Fournier’s gangrene, with time to surgical debridement being a principal factor in determining mortality. In a retrospective study of 72 Fournier’s gangrene cases, Kabay et al. found that non-survivors had significantly increased time intervals from onset of symptoms to first surgical intervention compared to survivors.^16^ Similarly, Yeniyol et al. reported that patients had significantly better outcomes with shorter times to consult.^17^ Since most infections are polymicrobial, management with empiric broad-spectrum antibiotics (covering gram positive, gram negative, and anaerobic organisms) is sufficient while awaiting culture results. Recommended regimens include vancomycin, clindamycin, and either a carbapenem or beta lactamase inhibitor.^18^ Due to the rapid pace at which antibiotic therapy and surgical intervention were initiated in our case, the patient ultimately survived and did well.

Additionally, the complex interplay of various hospital services was key in ensuring good outcome in the patient’s recovery. After the patient underwent surgical debridement, he was admitted under the care of multiple hospital teams including internal medicine, urology, ID, plastic surgery, and interventional radiology. There were multiple factors that initially complicated the patient’s hospital course, including the presence of multidrug-resistant organisms and poor wound healing. Furthermore, the patient had been off dialysis and immunosuppression prior to ED arrival, resulting in renal failure during his admission. With reinitiation of dialysis, local wound care, and multiple courses of antibiotics administered during the admission, the patient was eventually discharged home in stable condition.

This case demonstrates an atypical presentation of Fournier’s gangrene, in which an initially distracting complaint of abdominal pain revealed an underlying necrotizing infection of the patient’s scrotum. It exemplifies the importance of rapid identification, initiation of broad-spectrum antibiotics, and timely coordination with a surgical service. The patient’s age, male status, history of diabetes, and immunosuppressive state were all risk factors for the development of Fournier’s gangrene. Given the rapid progression of this infection, it is important for physicians to recognize vulnerable populations at risk and to have a low threshold for initiating aggressive treatment for this life-threatening condition.

## Supplementary Information






