# Supplementary figures and images for: A Case Report of an Atypical Presentation of Fournier’s Gangrene
Source: J Educ Teach Emerg Med. 2026 Jan 31;11(1):V9–V14. doi: 10.5070/M5.52203 (PMC12880891; doi:10.5070/M5.52203)

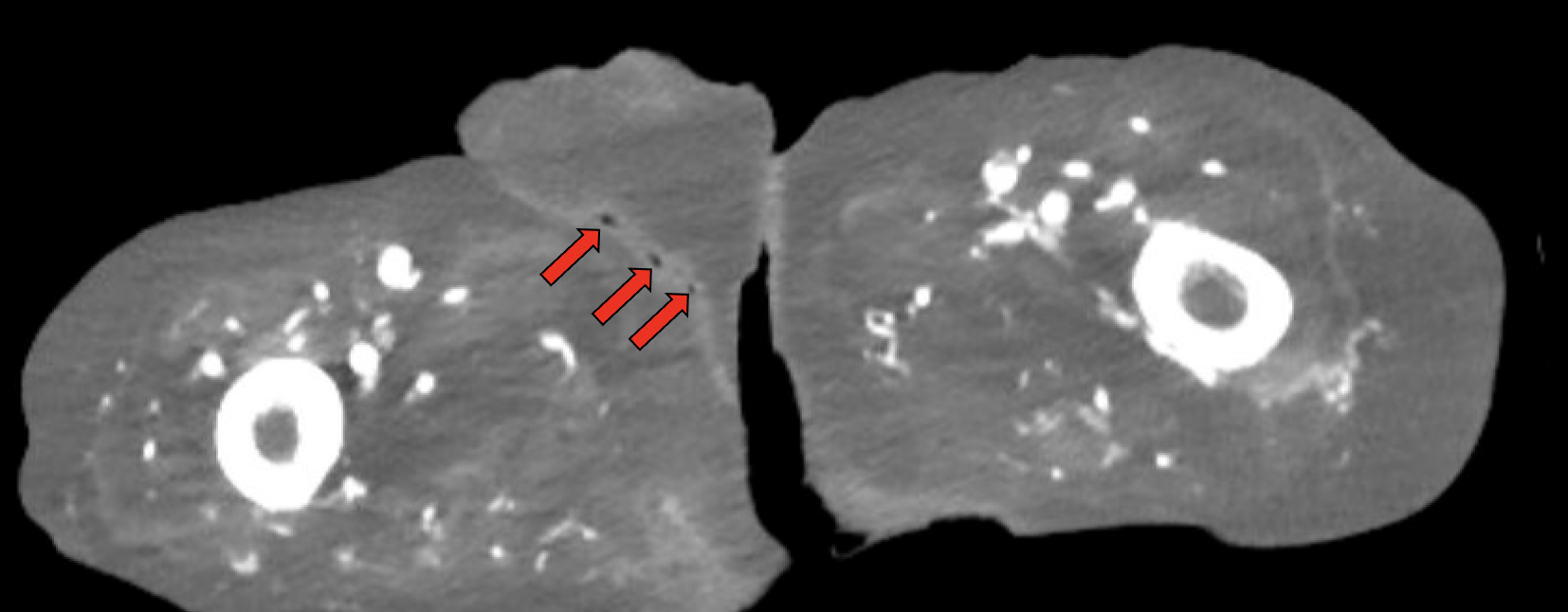

Supplement: Supplementary file 1 [file 11-1-V9-Supp1.png]

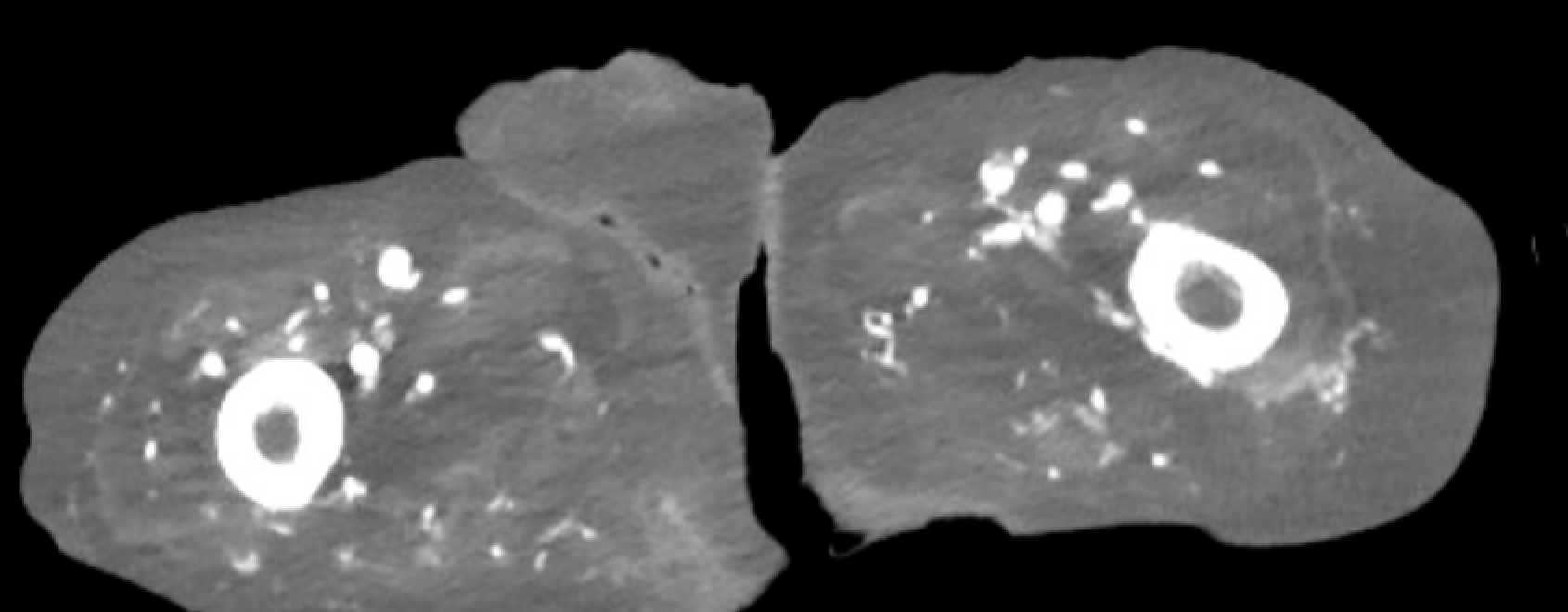

Supplement: Supplementary file 2 [file 11-1-V9-Supp2.png]

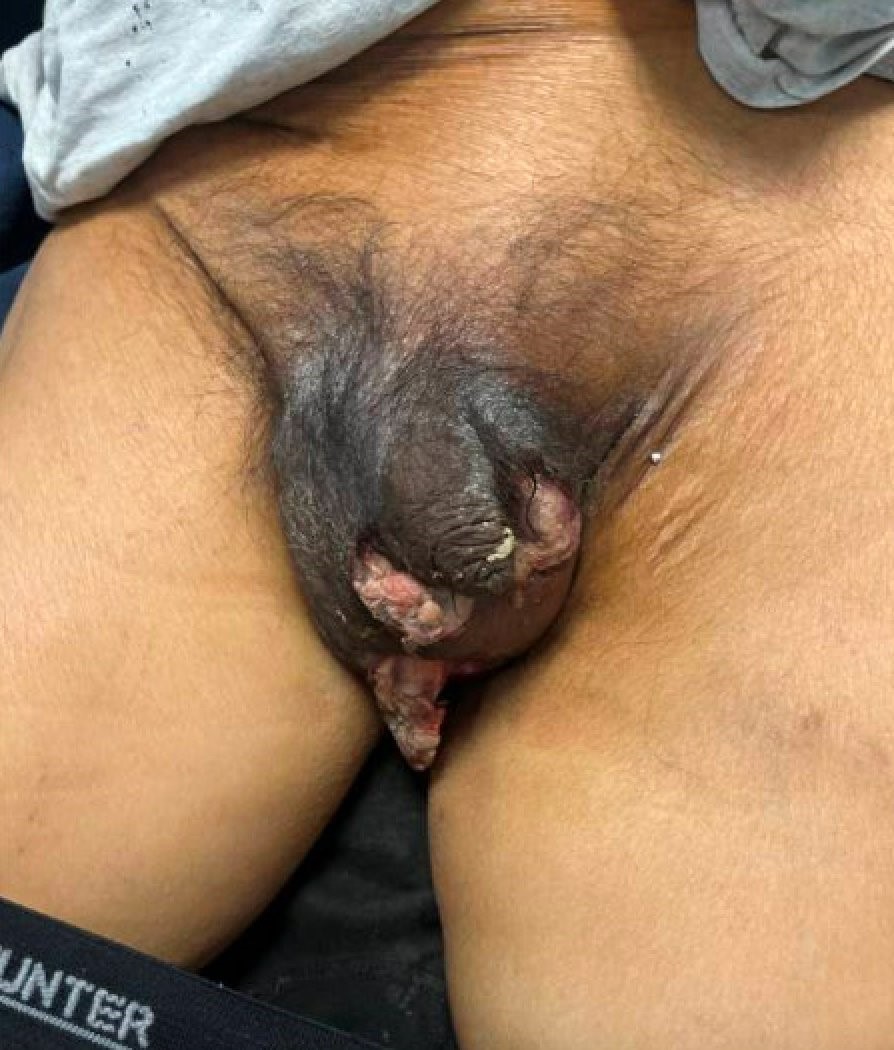

Supplement: Supplementary file 3 [file 11-1-V9-Supp3.jpg]
